# TRPC1‐mediated Ca^2+^ signaling enhances intestinal epithelial restitution by increasing α4 association with PP2Ac after wounding

**DOI:** 10.14814/phy2.14864

**Published:** 2021-05-15

**Authors:** Navneeta Rathor, Hee Kyoung Chung, Jia‐Le Song, Shelley R. Wang, Jian‐Ying Wang, Jaladanki N. Rao

**Affiliations:** ^1^ Cell Biology Group, Department of Surgery University of Maryland School of Medicine Baltimore MD USA; ^2^ Baltimore Veterans Affairs Medical Center Baltimore MD USA; ^3^ Department of Pathology University of Maryland School of Medicine Baltimore MD USA

**Keywords:** cell migration, epithelial restitution, IEC‐6 cells, mucosal injury, ornithine decarboxylase, polyamines, TRPC1

## Abstract

Gut epithelial restitution after superficial wounding is an important repair modality regulated by numerous factors including Ca^2+^ signaling and cellular polyamines. Transient receptor potential canonical‐1 (TRPC1) functions as a store‐operated Ca^2+^ channel in intestinal epithelial cells (IECs) and its activation increases epithelial restitution by inducing Ca^2+^ influx after acute injury. α4 is a multiple functional protein and implicated in many aspects of cell functions by modulating protein phosphatase 2A (PP2A) stability and activity. Here we show that the clonal populations of IECs stably expressing TRPC1 (IEC‐TRPC1) exhibited increased levels of α4 and PP2A catalytic subunit (PP2Ac) and that TRPC1 promoted intestinal epithelial restitution by increasing α4/PP2Ac association. The levels of α4 and PP2Ac proteins increased significantly in stable IEC‐TRPC1 cells and this induction in α4/PP2Ac complexes was accompanied by an increase in IEC migration after wounding. α4 silencing by transfection with siRNA targeting α4 (siα4) or PP2Ac silencing destabilized α4/PP2Ac complexes in stable IEC‐TRPC1 cells and repressed cell migration over the wounded area. Increasing the levels of cellular polyamines by stable transfection with the *Odc* gene stimulated α4 and PP2Ac expression and enhanced their association, thus also promoting epithelial restitution after wounding. In contrast, depletion of cellular polyamines by treatment with α‐difluoromethylornithine reduced α4/PP2Ac complexes and repressed cell migration. Ectopic overexpression of α4 partially rescued rapid epithelial repair in polyamine‐deficient cells. These results indicate that activation of TRPC1‐mediated Ca^2+^ signaling enhances cell migration primarily by increasing α4/PP2Ac associations after wounding and this pathway is tightly regulated by cellular polyamines.

## 1 INTRODUCTION

The gastrointestinal (GI) epithelium is exposed to a wide array of luminal noxious substances and microbiota, while acute mucosal injury occurs commonly in critical pathologies (Guo et al., 2002, 2003; Liu et al., 2017). Early mucosal restitution occurs as a consequence of intestinal epithelial cell (IEC) migration to reseal superficial wounds, a process independent of cell proliferation (Aihara & Montrose, 2014; Dignass et al., 1994). This early rapid re‐epithelialization after acute injury is a primary repair modality in the GI tract and is regulated by multiple factors, but its exact mechanism at the cellular and molecular levels remains largely unknown (Rao et al., 2001, 2008; Rathor, Chung, et al., 2014). Our previous studies and others show that cytosolic free Ca^2+^ ([Ca^2+^]_cyt_) plays a critical role in regulating epithelial restitution after acute injury and that Ca^2+^ entry due to store depletion contributes to the sustained increase in [Ca^2+^]_cyt_ and the refilling of Ca^2+^ into the stores (Rao et al., 2008, 2012, 2020; Wang et al., 2017). The canonical transient receptor potential channel 1 (TRPC1) functions as a store‐operated Ca^2+^ channel (SOC) in IECs and is absolutely required for rapid epithelial restitution after acute injury (Rao et al., 2006). Recently, it has been reported that TRPC1‐mediated Ca^2+^ influx stimulates epithelial restitution by activating caveolin 1 (Cav1) and RhoA signaling pathways (Chung et al., 2015; Rathor, Zhuang, et al., 2014).

Protein phosphatase 2A (PP2A)‐associated protein α4 (also named as immunoglobulin‐binding protein) has multiple functions and is implicated in a variety of cellular processes (Basu, 2011; Kong et al., 2007). α4 was initially identified as a component of receptor signal transduction complexes in mammalian lymphocytes, but it was later determined to be broadly expressed (Inui et al., 1995; LeNoue‐Newton et al., 2016; McConnell et al., 2010). α4 forms a stable complex with the catalytic (C) subunit of serine/threonine PP2A (PP2Ac) or with PP4 and PP6, which collectively account for many cellular phosphatase activities (Herzog et al., 2012; Kong et al., ,^2004^, ^2009^; Sents et al., 2013). Notably, PP2A heterotrimers are relatively unstable in response to stressful environments such as heat shock, DNA damage, and nutrient withdrawal, whereas overexpression of α4 results in a faster recovery of PP2A activity (Kong et al., 2009; Sents et al., 2013). The ability of α4 to protect PP2A from proteasomal‐mediated degradation is dependent on the integrity of its PP2A‐binding domain and C‐terminal domain which recruit microtubule‐associated E3 ubiquitin ligase and Mid1 (LeNoue‐Newton et al., 2011; McConnell et al., 2010; Watkins et al., 2012). Recently, α4/PP2Ac interaction is shown to regulate cell motility and be involved in activation of mTOR signaling pathways (Fielhaber et al., 2009; Wang & Jiang, 2003). The fibroblasts lacking α4 exhibit impaired ability to spread and migrate, but forced expression of the *α4* gene promotes cell spreading and motility (Kong et al., 2007). The level of α4 increases significantly in several cancers and transformed cells (Chen et al., 2011; LeNoue‐Newton et al., 2016) and this change affects apoptosis, proliferation, and migration in cancer cells (Kong et al., 2007; LeNoue‐Newton et al., 2016). We have recently found that intestinal epithelial tissue‐specific α4 ablation in mice disrupts intestinal mucosal maturation and reduces IEC migration along the crypt‐villus axis in the small intestine. The α4‐deficient intestinal epithelium also displays gut barrier dysfunction (Chung et al., 2018).

The purpose of the present study was to determine the possibility that TRPC1‐mediated Ca^2+^ signaling regulates IEC migration by altering α4/PP2Ac association after wounding. First, we examined the expression patterns of α4 and PP2Ac and their physical interactions in stable IEC‐TRPC1 cells. Second, we examined whether silencing α4 or PP2Ac inhibits epithelial restitution in IECs overexpressing TRPC1. Finally, we determined whether α4/PP2Ac interactions are regulated by cellular polyamines. Our results indicate that α4/PP2Ac associations are enhanced by activating TRPC1‐mediated Ca^2+^ signaling and this induction promotes early intestinal epithelial restitution.

## 2 MATERIALS AND METHODS

### 2.1 Chemicals and cell culture

Disposable culture ware was purchased from Corning Glass Works. Tissue culture media, LipofectAMINE 2000, and dialyzed fetal bovine serum (FBS) were obtained from Invitrogen, and biochemicals were obtained from Sigma. The affinity‐purified mouse monoclonal antibodies against α4 (Catalog#5699) and PP2Ac (Catalog#2259) were purchased from Cell Signaling Technologies and the TRPC1 antibody from Santa Cruz Biotechnology (Catalog#sc‐133076). The actin antibody that recognizes all isoforms (α, β, and γ; Cat#CP‐01)) was purchased from EMD Millipore. The secondary antibodies conjugated to horseradish peroxidase (goat anti‐rabbit IgG‐HRP, cat#sc‐2004; goat anti‐mouse IgG HRP, cat#sc‐2005) were purchased from Santa Cruz Biotechnology. L‐α‐difluoromethylornithine (DFMO) was from Genzyme. Human *α4* cDNA was purchased from OriGene Technologies (Catalog#RC200387). The IEC‐6 cell line was purchased from the American Type Culture Collection (ATCC) at passage 13. IEC‐6 cells were derived from normal rat intestinal crypt cells and were developed and characterized by Quaroni et al. (1979). Stock cells were maintained in T‐150 flasks in Dulbecco's modified Eagle medium (DMEM) supplemented with 5% heat‐inactivated FBS, 10 μg/ml insulin, and 50 μg/ml gentamicin sulfate. Flasks were incubated at 37^○^C in a humidified atmosphere of 90% air‐10% CO_2_, and *passages 15–20* were used in the experiments.

### 2.2 Plasmid construction and transfection

The full‐length cDNA of human TRPC1 (~3.8 kb) was inserted into the Not1 and Apa1 sites of expression vector pcDNA3.0(+) (Invitrogen) with the cytomegalovirus promoter (pcDNA‐TRPC1) (Rao et al., 2006). The IEC‐6 cells were transfected with the pcDNA‐TRPC1 or pcDNA3.0(+) vectors containing no TRPC1 cDNA (Null) by using the LipofectAMINE kit and performed as recommended by the manufacturer (Invitrogen). After the 3‐h period of incubation, the transfection medium was replaced by the standard growth medium containing 5% FBS for 2 days before exposure to the selection medium. These transfected cells were selected for TRPC1 integration by incubation with the selection medium containing 0.6 mg/ml of G418, and clones resistant to the selection medium were isolated, cultured, and screened for TRPC1 expression as described in our previous publications (Chung et al., 2015; Rao et al., 2006). Similarly, stable ODC‐transfected IEC‐6 cells (IEC‐ODC) were developed and characterized as described (Rathor, Chung, et al., 2014; Wang et al., 2010) and cultured in DMEM medium used for growing IEC‐6 cells.

### 2.3 Polyamine depletion studies

We examined the effect of polyamine depletion on the α4 and PP2Ac expression in IEC‐6 cells. IEC‐6 cells were initially grown in DMEM containing 5% dFBS in the presence of either 5 mM DFMO alone or DFMO plus 10 μM putrescine for 4 days. The dishes were placed on ice, the monolayers were washed three times with ice‐cold Dulbecco's PBS (D‐PBS), and then different solutions were added according to the assays to be performed. In addition, the effect of ectopic overexpression of α4 on cell migration in the presence of DFMO was also examined in IECs.

### 2.4 RNA interference

The siRNA that was designed to specifically cleave α4 and PP2Ac mRNAs (siα4 or siPP2Ac) was synthesized (ON‐TARGETplus SMARTpool) and purchased from Dharmacon. Scrambled control siRNA (C‐siRNA), without sequence homology to any known genes, was used as the control. For each 60‐mm cell culture dish, 20 µl of the 5 µM stock siα4 or siPP2Ac or C‐siRNA was mixed with 500 µl of Opti‐MEM medium (Invitrogen). This mixture was gently added to a solution containing 6 µl of LipofectAMINE 2000 in 500 µl of Opti‐MEM. The solution was incubated for 15 min at room temperature and gently overlaid onto monolayers of cells in 3 ml of medium, and cells were harvested for various assays after 48‐h incubation.

### 2.5 Immunoprecipitation and Western blotting analysis

Cell samples, dissolved in ice‐cold RIPA‐buffer, were sonicated and centrifuged at 4°C and the supernatants were collected for immunoprecipitation (IP). Equal amounts of proteins (300 μg) for each sample were incubated with the specific antibody against α4 or PP2Ac (4 μg) at 4°C for 3 h, and protein A/G‐PLUS‐Agarose was added and incubated overnight at 4°C. The precipitates were washed five times with ice‐cold Tris‐buffered saline (TBS), and the beads were resuspended in SDS sample buffer. For immunoblotting, samples were subjected to electrophoresis on PAGE gels described previously (Jiang et al., 2019; Rathor, Chung, et al., 2014; Zhang et al., 2020). After the transfer of protein onto nitrocellulose membranes, the membranes were incubated for 1 h in 5% non‐fat dry milk in 1× TBS‐T buffer (0.1% Tween‐20). Immunologic evaluation was then performed overnight at 4°C in 5% non‐fat dry milk/TBS‐T buffer containing a specific antibody against α4 or PP2Ac. The membranes were subsequently washed with 1× TBS‐T and incubated with the secondary antibodies conjugated with horseradish peroxidase for 1 h at room temperature. The immunocomplexes on the membranes were reacted for 1 min with Chemiluminiscence Reagent (NEL‐100 DuPont NEN). All antibodies utilized in this paper were thoroughly validated for species specificity. Antibody dilutions used in this studies were; α4 (First Ab 1:1000; second AB 1:2000); PP2Ac (First Ab 1:2000; second Ab 1:2000); TRPC1 (First Ab 1:500; second Ab 1:1000); and Actin (First Ab 1:1000; second Ab 1:2500).

### 2.6 Measurement of cell migration

Migration assays were carried out in an in vitro epithelial injury model as described in our earlier publications (Chung et al., 2015; Rao et al., 1999, 2006, 2008; Rathor, Chung, et al., 2014). Cells were plated at 6.25 × 10^4^/cm^2^ in DMEM containing FBS on 60‐mm dishes thinly coated with Matrigel according to the manufacturer's instructions (BD Biosciences, Bedford, MA) and were incubated as described for stock cultures. Cells were fed on day 2, and cell migration was assayed on day 4. To initiate migration, the cell layer was scratched with a single edge razor blade cut to ~27 mm in length. The scratch was made over the diameter of the dish and extended over an area 7–10 mm wide. The migrating cells in six contiguous 0.1‐mm squares were counted at ×100 magnification beginning at the scratch line and extending as far out as the cells had migrated. All experiments were carried out independently 3–5 times, and the results were reported as number of migrating cells per millimeter of scratch.

### 2.7 Immunofluorescence staining

Immunofluorescence staining was performed as described previously (Rathor, Zhuang, et al., 2014). After IECs were grown initially for 2 days, they were fixed using 4% formaldehyde, and the samples were incubated overnight at 4°C with primary antibody against α4 and PP2Ac diluted 1:100 in blocking buffer and then incubated with secondary antibodies conjugated with Cy5 and Alexa Fluor‐488 (Molecular Probes) to detect subcellular localization respectively for 1 h at room temperature. After rinsing, slides were incubated with DAPI (1:5000) (Molecular Probes) for 10 min to stain nuclei, rinsed again, mounted, and viewed through a Nikon Eclipse Ti. Images were processed using PhotoShop software (Adobe).

### 2.8 Statistical analysis

All data for migration experiments are expressed as means±SEM from six dishes in each experiment and independently repeated three times (*n* = 3). IP and immunoblotting analyses were repeated three times (*n* = 3). The significance of the difference between means was determined by one‐way ANOVA with Dunnett's post hoc test (GraphPad Instat Prism 5). The level of significance was determined using the Duncan's multiple‐range test (Harter, 1960) and values of *p* < 0.05 were considered statistically significant.

## 3 RESULTS

### 3.1 Induced α4/PP2Ac association in stable IEC‐TRPC1 cells

**FIGURE 1:**
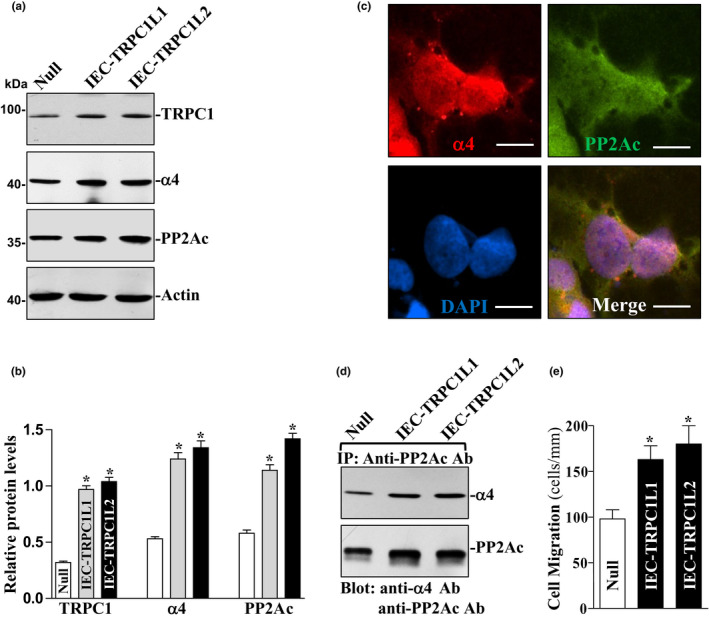
Levels of α4 and PP2Ac proteins, their physical interactions, and cell migration in the stable TRPC1‐transfected IEC (IEC‐TRPC1) cells. (a) representative immunoblots of TRPC1, α4 and PP2Ac. Levels of TRPC1, α4 and PP2Ac were examined by Western blot analysis, and actin immunoblotting was performed as an internal control for equal loading in two different clonal populations (L1&L2). Three separate experiments were performed that showed similar results. (b) quantitative analysis of western immunoblots by densitometry that were corrected for actin loading from cells described in (a). Values are means ± SEM; *p < 0.05 compared with null. (c) subcellular distribution of α4 and PP2Ac in Caco‐2 cells. After cells were initially grown for 48 h, immunostaining was performed. Representative images showing α4 and PP2Ac co‐localization. Scale bar, 50 μM. Magnification ×200; red: α4; green: PP2Ac; blue: DAPI (nuclei). (d) levels of α4/PP2Ac complex as measured by immunoprecipitation (IP) by the anti‐PP2Ac antibody from cells described in (a). After whole cell lysates were IP by the anti‐PP2Ac antibody, precipitates were separated by SDS‐PAGE and blotted by using anti‐ α4 or anti‐PP2Ac antibody. (e) summarized data of cell migration after wounding in cells described in (a). Cell migration was assayed 6 h after removing part of the monolayer. Data are means ± SE from 6 dishes. *p < 0.05 compared with null

To determine if TRPC1‐mediated Ca^2+^ influx increases α4/PP2Ac association, basal levels of α4 and PP2Ac and their interactions were examined in stable IEC‐TRPC1 cells. IEC‐TRPC1 cells highly expressed TRPC1 (by ~5‐fold) and displayed a significant increase in Ca^2+^ influx (by ~2‐fold) after depletion of store Ca^2+^ by cyclopiazonic acid, similar to our previous studies (Chung et al., 2015; Rao et al., 2006, 2010). As shown in Figure 1a, levels of TRPC1, α4, and PP2Ac proteins increased in two clones (Cs) of stable IEC‐TRPC1 cells compared to those observed in parental IEC‐6 cells transfected with an empty vector (Null). The levels of α4 and PP2Ac in C1 and C2 of IEC‐TRPC1 cells were ~2.3‐fold of the value of null cells (Figure 1b). To examine the cellular distribution of α4 and PP2Ac, immunofluorescence staining was performed and showed co‐localization of α4 and PP2Ac in the cytoplasm (Figure 1c). To further verify the association of α4 with PP2Ac, whole cell lysates were immunoprecipitated (IP) with the anti‐PP2Ac antibody and the precipitates were examined by Western blot analysis using the antibody against α4 or PP2Ac. IP of PP2Ac resulted in co‐IP of α4 and PP2Ac in null and stable IEC‐TRPC1 cells, but the levels of α4/PP2Ac complexes in IEC‐TRPC1 cells were higher than those observed in null cells (Figure 1d). We also used IgG as a negative control in IP assays and found that incubation with IgG in the same conditions did not pull down either α4 or PP2Ac (data not shown). Like the induction of α4/PP2Ac association, IEC migration over the wounded area also increased significantly in IEC‐TRPC1 cells compared with null cells after injury (Figure 1e). These results indicate that activated TRPC1‐mediated Ca^2+^ signaling increases the levels of α4/PP2Ac complex, which is associated with an increase in cell migration after wounding.

### 3.2 Silencing α4 or PP2Ac reduces α4/PP2Ac complexes and represses epithelial restitution after wounding in IEC‐TRPC1 cells

**FIGURE 2:**
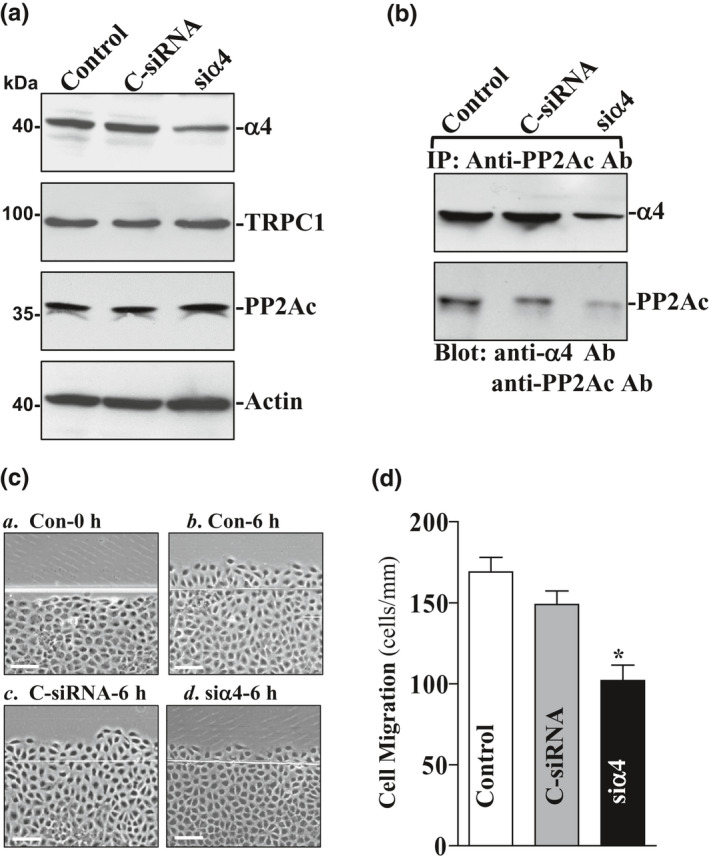
Effect of α4 silencing on the levels of α4/PP2Ac complex and cell migration. (a) representative immunoblots of α4, TRPC1, and PP2Ac in IEC‐TRPC1L1 cells transfected with siα4 or C‐siRNA for 48 h. (b) levels of α4/PP2Ac complex in cells described in (a). Levels of α4 and PP2Ac in IP complex were measured by Western blot analysis using specific antibodies. (c) images of cell migration after wounding: (a) 0 h after wounding; (b) 6 h after wounding; (c) 6 h after wounding in cells transfected with C‐siRNA for 48 h; and (d) 6 h after wounding in cells transfected with siα4 for 48 h. (d) summarized data of cell migration after wounding in cells described in (a). Values are means ± SE from 6 dishes. *p < 0.05 compared with controls and cells transfected with C‐siRNA

In this study, specific siRNA targeting α4 mRNA (siα4) was used to cleave rat α4 mRNA and inhibit α4 expression. Our preliminary study demonstrated that >85% of stable IEC‐TRPC1 cells were positive 48 h after transfection with fluorescent FITC‐conjugated siα4. We have also tested the transfection efficiency at other time points such as 24 and 72 h and found that 48 h post‐transfection appeared optimal to carry out the experiments. As shown in Figure 2a, transfection with siα4 for 48 h decreased α4 levels by ~80%. To determine the specificity of siα4 used in this study, we reprobed the membrane with either anti‐TRPC1 or anti‐PP2Ac antibody and showed that levels of TRPC1 and PP2Ac proteins were not affected when cells were transfected with siα4. However, α4 silencing by transfection with siα4 decreased α4/PP2Ac complexes (by ~65%) when compared with those observed in control cells and cells transfected with control siRNA (C‐siRNA) (Figure 2b). Furthermore, decreased α4/PP2Ac association by α4 silencing suppressed cell migration (by ~40%) after wounding (Figure 2c and d). Neither α4 expression nor cell migration was decreased by transfection with C‐siRNA. In addition, transfection with siα4 or C‐siRNA did not alter cell viability as measured by Trypan blue staining (data not shown).

**FIGURE 3:**
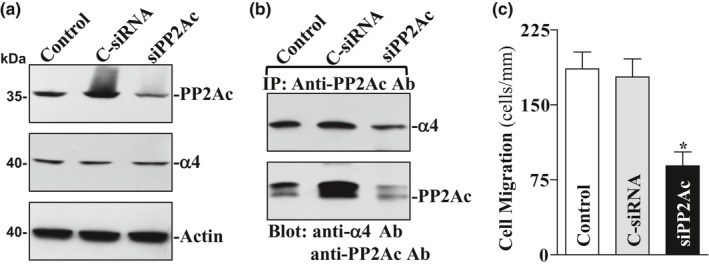
Effect of PP2Ac silencing on the levels of α4/PP2Ac complex and cell migration. (a) representative immunoblots of PP2Ac and α4 in IEC‐TRPC1L1 cells transfected with siPP2Ac or C‐siRNA for 48 h. (b) levels of α4/PP2Ac complex as measured by IP assay in cells described in (a). (c) summarized data of cell migration after wounding in cells described in (a) Values are means ± SE from 6 dishes. *p < 0.05 compared with controls and cells transfected with C‐siRNA

To examine the effect of PP2Ac silencing on cell migration, specific siRNA targeting PP2Ac mRNA (siPP2Ac) was used to inhibit PP2Ac expression in IEC‐TRPC1 cells. As shown in Figure 3a, transfection with siPP2Ac for 48 h decreased PP2Ac levels by ~80%, although it failed to affect the levels of α4 protein. As expected, PP2Ac silencing by transfection with siPP2Ac also decreased α4/PP2Ac complexes (by ~75%) when compared with those observed in control cells and cells transfected with control siRNA (C‐siRNA) (Figure 3b). Consistently, decreased α4/PP2Ac association by PP2Ac silencing also suppressed cell migration (by ~60%) after wounding (Figure 3c). These findings indicate that α4/PP2Ac association is necessary for the stimulation of cell migration after wounding in IEC‐TRPC1 cells.

### 3.3 Elevation of cellular polyamine levels enhances expression of α4 and PP2Ac and stimulates cell migration

**FIGURE 4:**
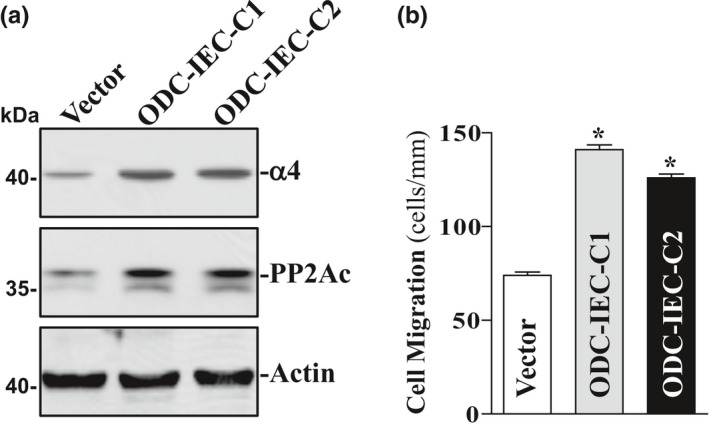
Changes in the levels of α4 and PP2Ac and cell migration after increasing the levels of cellular polyamines. (a) changes in α4 and PP2Ac protein levels in clonal (c) populations of ODC‐IEC cells and control cells (Vector). IEC‐6 cells were infected with either the retroviral vector containing the sequence encoding mouse ODC cDNA or control retroviral vector lacking ODC cDNA. Clones resistant to the selection medium containing 0.6 mg/ml G418 were isolated and screened for ODC expression. Levels of α4 and PP2Ac proteins were measured by Western blot analysis, and equal loading was monitored by actin immunoblotting. Three separate experiments were performed that showed similar results. (b) summarized data showing cell migration 6 h after wounding in cells described in (a). Values are means ± SEM of data from 6 dishes. *p < 0.05 compared with Vector cells

Polyamines, including spermidine, spermine, and their precursor putrescine, are organic cations found in all eukaryotic cells and act as biological regulators of gut mucosal repair after acute injury in vivo as well as in vitro (Igarashi & Kashiwagi, 2019; Liu et al., 2009; Rao et al., 2020; Seiler & Raul, 2007; Tabor & Tabor, 1984). Polyamine biosynthesis is predominantly dependent on activity of the key rate‐limiting enzymes ornithine decarboxylase (ODC) (Rao et al., 2020), and induced levels of cellular polyamines promote intestinal mucosal repair primarily by enhancing early epithelial restitution after wounding (Rao et al., 1999, 2001; Rathor, Chung, et al., 2014). To determine the effect of cellular polyamines on α4 and PP2Ac expression, two clonal populations of IECs stably overexpressing ODC (ODC‐IEC) (Ouyang et al., 2015; Rathor, Chung, et al., 2014; Zou et al., 2012) were used in this study. These stable ODC‐IECs exhibited very high levels of ODC protein and greater than 50‐fold increase in ODC enzyme activity (Zou et al., 2012). Consistently, the levels of putrescine, spermidine, and spermine in ODC‐IEC cells were increased by ~12‐fold, ~2‐fold, and ~25%, respectively, when compared with cells transfected with the control vector lacking *Odc* cDNA, as described in our previous studies (Ouyang et al., 2015; Wang et al., 2010; Zou et al., 2012). As shown in Figure 4, there was a substantial increase in the expression of α4 and PP2Ac in ODC‐IEC cells. The levels of α4 protein increased by >3‐fold in stable ODC‐IEC cells as compared with those observed in cells transfected with the control vector. The levels of PP2Ac protein increased by ~2‐fold. The increased expression of α4 and PP2Ac in ODC‐transfected cells was not simply due to clonal variation, since two stable clones, ODC‐IEC‐C1 and ODC‐IEC‐C2, showed similar responses. Increasing the levels of cellular polyamines by ectopic ODC overexpression also increased the epithelial restitution, as indicated by an increase in cell migration over the wounded area in stable ODC‐IECs (Figure 4b). Similar results have been reported in our previous publications (Ouyang et al., 2015; Rathor, Chung, et al., 2014; Wang et al., 2010). These findings indicate that increased levels of α4 and PP2Ac by polyamines stimulate epithelial restitution.

### 3.4 Polyamine depletion decreases levels of α4 and PP2Ac and repress cell migration

**FIGURE 5:**
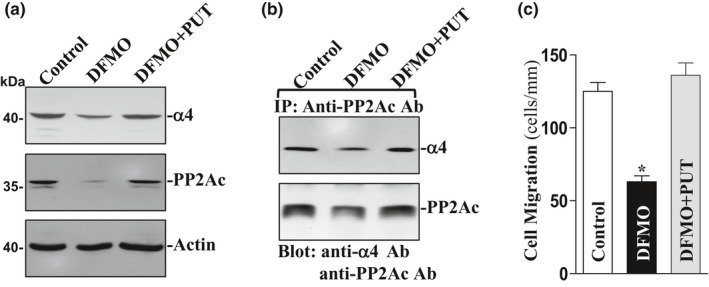
Changes in the levels of α4 and PP2Ac proteins, their association, and cell migration after polyamine depletion. (a) representative immunoblots of α4 and PP2Ac proteins. IEC‐6 cells were grown in the medium containing DFMO (5 mM) alone or DFMO plus putrescine (Put, 10 μM) for 4 days. Levels of α4 and PP2Ac proteins were measured by Western blot analysis, and equal loading was monitored by actin immunoblotting. Three separate experiments were performed that showed similar results. (b) levels of α4 and PP2Ac proteins in the complex IP by the anti‐PP2Ac Ab from the samples described in (a). (c) summarized data showing cell migration 6 h after wounding in cells described in (a). Values are means ± SEM of data from 6 dishes. *p < 0.05 compared with control cells or cells treated with DFMO plus Put

**FIGURE 6:**
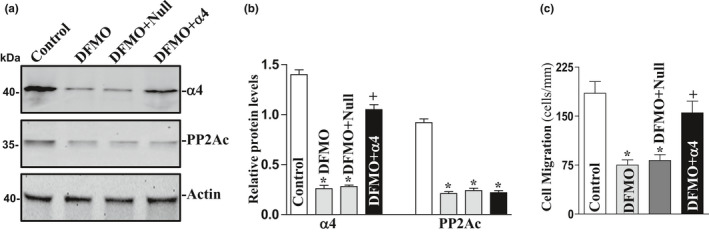
Effect of α4 overexpression on cell migration in polyamine‐deficient cells. (a) representative immunoblots of α4 and PP2Ac. IECs were exposed to 5 mM DFMO for 2 days and then transfected with either α4 expression vector or empty vector (Null). By 48 h after transfection in the presence of DFMO, levels of α4 and PP2Ac protein levels were measured by Western blot analysis. Three experiments were performed that showed similar results. (b) quantitative analysis of western immunoblots by densitometry that were corrected for actin loading from cells described in (a). Values are means ± SEM; *p < 0.05 compared with control cells. ^+^ *p* < 0.05 versus DFMO alone. (c) summarized data showing cell migration 6 h after wounding in cells described in (a). Values are means ± SEM of data from 6 dishes. *p < 0.05 compared with control cells. ^+^ *p* < 0.05 versus DFMO alone

To further determine the role of endogenous polyamines in the regulation of α4/PP2Ac interaction, IEC‐6 cells were exposed to 5 mM DFMO (a specific inhibitor of polyamine biosynthesis) to deplete cellular polyamines. As reported previously (Guo et al., 2002; Rao et al., 1999), exposure to DFMO for 4 days completely depleted putrescine and spermidine and also substantially decreased spermine content. Polyamine depletion by DFMO inhibited expression of α4 and PP2Ac, and the levels of α4 and PP2Ac proteins in polyamine‐deficient cells were decreased by ~60% and 95%, respectively (Figure 5a). Consistently, decreased α4/PP2Ac complexes by polyamine depletion inhibited cell migration (Figure 5b and c). Exogenous polyamine putrescine (10 μM) given together with DFMO not only prevented the decreased levels of the α4/PP2Ac complex but also restored cell migration to near normal level after wounding. Moreover, ectopic overexpression of α4 also partially prevented inhibition of cell migration induced by polyamine depletion (Figure 6). In this study, cells were exposed to DFMO for 2 days and then transfected with α4 expression vector for 48 h in the presence of DFMO. Ectopic overexpression of α4 significantly rescued cell migration after wounding in polyamine‐deficient cells (Figure 6c). The number of cells migrating over the denuded area increased by ~30% in polyamine‐deficient cells transfected with α4 expression vector compared to DFMO‐treated cells transfected with empty vector (null). Taken together, these results indicate that polyamine depletion inhibits intestinal epithelial restitution, at least partially, by downregulating α4 and PP2Ac expression and their interaction.

## 4 DISCUSSION

In response to acute mucosal injury in the gut, damaged IECs are sloughed and remaining viable cells migrate to cover the wounded area rapidly, a process named as epithelial restitution (Dignass et al., 1994; Nusrat et al., 1992; Rao & Wang, 2016; Silen & Ito, 1985). This early epithelial restitution is crucial for maintenance of the intestinal epithelial integrity under physiological and pathological conditions, but its exact mechanism remains largely unknown. Our previous studies have shown that TRPC1 functions as a SOC channel in IECs and plays an important role in early epithelial restitution by increasing Ca^2+^ influx (Chung et al., 2015; Rao et al., 2006, 2010; Rathor, Zhuang, et al., 2014). The current study provides new evidence that increased association of α4 with PP2Ac is crucial for TRPC1‐mediated stimulation of cell migration after wounding, thereby advancing our understanding of the mechanism underlying early epithelial restitution. α4 directly interacted with PP2Ac in IECs and this interaction was increased by TRPC1‐mediated Ca^2+^ signaling. Induced α4/PP2Ac complex in IEC‐TRPC1 cells is necessary for increasing cell migration after wounding. Moreover, our findings also reveal that polyamines positively modulate expression of α4 and PP2Ac and enhance α4/PP2Ac association.

The results reported herein clearly indicate that the increased levels of α4 and PP2Ac in stable IEC‐TRPC1 cells were associated with an increase in cell migration after wounding. Increased levels of α4/PP2Ac complexes are essential for stimulation of epithelial restitution in IEC‐TRPC1 cells, since silencing α4 or PP2Ac inhibited formation of α4/PP2Ac association and suppressed cell migration after wounding. It has been reported that α4 forms a stable complex with the catalytic (C) subunit of serine/threonine PP2A or with PP4 and PP6 and that PP2A heterotrimers are relatively unstable in response to stressful environments. However, overexpression of α4 results in a faster recovery of PP2A activity (Janssens & Rebollo, 2012; Kong et al., 2009; LeNoue‐Newton et al., 2016). Our previous studies have shown that the levels of α4 protein increased remarkably 1 h after wounding and this induction in α4 levels was associated with an increase in the cellular abundance of PP2A. Further studies reveal that α4 is required for intestinal mucosal maturation, whereas α4 deletion in IECs alters proliferation, migration, apoptosis, and cell‐to‐cell interaction in mice (Chung et al., 2018). In another study, Kong et al defined a novel role of α4 in the control of cell spreading and migration in fibroblasts (Kong et al., 2007). The levels of GTP‐bound Rac1 decrease in fibroblasts lacking α4, whereas ectopically expressed α4 restores Rac1 activity and promotes cell migration (Kong et al., 2007). Moreover, other evidence also exists supporting a pro‐proliferative influence of α4. For examples, cell proliferation is stimulated by ectopic α4 overexpression in several cancer cells but inhibited by α4 silencing (Chen et al., 2011; Liu et al., 2014). Although the expression patterns of α4 and PP2Ac in damaged IG mucosa in vivo remain unknown, our ongoing studies using gut mucosal injury model in mice will provide necessary information in the future.

The results presented here also show that increasing cellular polyamines by overexpressing ODC increased the protein levels α4 and PP2Ac and enhanced their association, thus stimulating cell migration. In contrast, depletion of cellular polyamines decreased levels of α4 and PP2Ac complexes and inhibited restitution. In polyamine‐deficient cells, overexpression of α4 partially rescued cell migration. Polyamines are potent regulators of gut mucosal repair (Rao et al., 2020; Timmons et al., 2012; Wang & Johnson, 1991), whereas inhibition of polyamine biosynthesis represses early epithelial repair by inactivating multiple signaling pathways including TRPC1/Ca^2+^ signaling (Chung et al., 2018; Rathor, Zhuang, et al., 2014). Polyamines enhance gut mucosal repair by regulating multiple signaling pathways including small GTP‐binding proteins such as RhoA and Rac1 (Chung et al., 2015; Rao et al., 2001, 2008), STIM1 signaling (Rao et al., 2010, 2012; Rathor, Zhuang, et al., 2014), and c‐Jun/PLC‐γ1 pathways (Rao et al., 2008; Wang et al., 2017). The current studies provide an additional evidence showing that increased levels of α4 and PP2Ac and their interaction also contribute to stimulation of gut epithelial restitution by polyamines.

In summary, our results indicate that activation of TRPC1‐mediated Ca^2+^ signaling in stable IEC‐TRPC1 cells induces expression of α4 and PP2Ac and thus increases the levels of α4/PP2Ac complex. The increased interaction of α4​ with PP2Ac is essential for stimulation of cell migration after wounding in IECs overexpressing TRPC1. Specific inhibition of α4 or PP2Ac prevents formation α4/PP2Ac complexes and represses cell migration after wounding. Moreover, increasing the levels of cellular polyamines also enhances expression of α4 and PP2Ac in IECs, whereas polyamine depletion decreases cellular α4 and PP2Ac abundances, suggesting that polyamines promote intestinal epithelial restitution at least partially by enhancing α4/PP2Ac complex. Together, our results indicate that increased α4/PP2Ac associations by TRPC1‐mediated Ca^2+^ signal and polyamines play an important role in the stimulation of intestinal epithelial restitution after wounding.

## CONFLICT OF INTEREST

None.
